# Changes in life satisfaction and leisure-time physical activity across retirement transition: the FIREA cohort study

**DOI:** 10.1007/s10433-025-00865-x

**Published:** 2025-06-17

**Authors:** Miika Tuominen, Säde Stenlund, Kristin Suorsa, Jaana Pentti, Jussi Vahtera, Tuija Leskinen, Pasi Koski, Sari Stenholm

**Affiliations:** 1 Department of Public Health, University of Turku and Turku University Hospital, Turku, Finland; 2 Centre for Population Health Research, University of Turku and Turku University Hospital, Turku, Finland; 3 Department of Social and Behavioral Sciences, Harvard T.H. Chan School of Public Health, Boston, MA 02115 USA; 4 Clinicum, Faculty of Medicine, University of Helsinki, Helsinki, Finland; 5 Department of Teacher Education, University of Turku, 26101 Rauma, Finland; 6 Research Services, University of Turku and Turku University Hospital, Turku, Finland

**Keywords:** Retirement, Physical activity, Leisure, Well-being, Life satisfaction, Aging

## Abstract

Retirement brings changes in daily life, which may have implications for life satisfaction and leisure-time physical activity (LTPA). This study sought to examine associations between concurrent changes in life satisfaction and LTPA across retirement, addressing gaps in understanding how life satisfaction influences physical activity in retirees. Study included 3535 public sector workers (83% women) from the Finnish Retirement and Aging study with annual surveys before and after retirement. Participants were categorized based on their Life Satisfaction Scale responses before and after retirement into Stable Low, Low-Increasing, Intermediate-Decreasing, Stable Intermediate, Intermediate-Increasing, High-Decreasing, and Stable High life satisfaction. Self-reported weekly LTPA during past 12 months was summarized as metabolic equivalent hours (METh/week). Generalized estimating equations were used to examine changes in LTPA across retirement transition among these groups, adjusting for sociodemographic and health-related factors. Compared to Stable Low, Low-Increasing life satisfaction was associated with increasing LTPA (mean difference 4.16 METh/week, 95%CI 0.85 to7.47). Compared to Stable Intermediate, Intermediate-Increasing life satisfaction was associated with increasing LTPA (mean difference 1.96 METh/week, 95%CI 0.03 to 3.88), while Intermediate-Decreasing life satisfaction was linked to decreasing LTPA (mean difference  −3 .79 METh/week, 95%CI −7.62 to 0.04). No differences were observed between Stable High and High-Decreasing life satisfaction. Changes in life satisfaction during retirement transition are associated with changes in LTPA, especially among those with low or intermediate life satisfaction before retirement. Further research is needed to assess, whether supporting life satisfaction in retirees could foster greater engagement in LTPA.

**Supplementary Information:**

The online version contains supplementary material available at 10.1007/s10433-025-00865-x.

## Introduction

Retirement transition represents an opportunity for increasing leisure-time physical activity (LTPA) at the gates of older age (Lane-cordova et al. 2021). Previous studies have reported that self-reported LTPA tends to increase after retirement transition (Xue et al. 2020), particularly moderate-intensity activities (Stenholm et al. 2016), such as recreational walking (Jones et al. 2018b). However, among people retiring on health- rather than age-based reasons, less favorable changes have been observed (Ding et al. 2016; Stenholm et al. 2016). Furthermore, even in studies where retirement is most likely to occur due to age-based reasons, the changes in LTPA seem to vary based on socioeconomical status (Vansweevelt et al. 2022), health (Jones et al. 2018a; Stenholm et al. 2016), and physical activity habits preceding retirement (Ter Hoeve et al. 2020). Gender, however, does not appear to moderate the association between retirement and LTPA with similar increases commonly observed among both men and women (Ding et al. 2016; Jones et al. 2018a; Sjösten et al. 2012; Stenholm et al. 2016; Tunney et al. 2023). Qualitative studies suggest that retirement transition may facilitate LTPA engagement through perceived increases in time and energy availability, re-evaluation of individual’s life goals and values, increased interest in health, and increased motivation for LTPA (Barnett et al. 2012; Beck et al. 2010; Kosteli et al. 2016; McDonald et al. 2015; Rai et al. 2020). On the other hand, retirement transition may also bring adaptation challenges associated with the loss of work-related roles, social contacts and daily routines, and the need for developing a satisfactory post-retirement lifestyle (van Solinge and Henkens 2008). These challenges, together with potentially co-occurring age-related decline in health and physical functioning, may hinder LTPA engagement, particularly among those less accustomed to including LTPA in their daily schedules before retirement (Barnett et al. 2012; Beck et al. 2010; Kosteli et al. 2016; McDonald et al. 2015; Rai et al. 2020). Thus, retirement transition should be viewed as a process requiring psychological adjustment, the success of which is likely to influence and be influenced by engagement in LTPA.

Adjustment to retirement is often assessed indirectly through changes in life satisfaction across retirement transition (Barbosa et al. 2016). Life satisfaction refers to the cognitive component of subjective well-being and is characterized as judgmental evaluation of one’s life as a whole (Diener 1984). Based on previous longitudinal studies, retirement transition tends to be associated with small increases in the overall levels of life satisfaction (Hansson et al. 2018; Prakash et al. 2022) with majority of participants reporting relatively high levels of life satisfaction both before and after retirement transition (Heybroek et al. 2015; Pinquart and Schindler 2007; Wang 2007). Yet, considerable heterogeneity has also been reported with low socioeconomical status, poor health, being unmarried, and younger retirement age associated with less favorable changes in life satisfaction across retirement transition (Heybroek et al. 2015; Pinquart & Schindler 2007; Wang 2007). Some studies suggest that lower levels or less favorable changes in life satisfaction might be more common among women (Heybroek et al. 2015; Pinquart and Schindler 2007), whereas others have observed higher increases in life satisfaction among women across retirement transition (Prakash et al. 2022). There are also studies suggesting that women in particular might benefit from retirement in terms of both improved physical and mental health with coinciding increases in physical activity engagement (Zhu 2016).

Life satisfaction and LTPA tend to be positively associated (Wiese et al. 2018) with some evidence indicating that this association might become stronger following retirement (Kuykendall et al. 2015). This suggests that LTPA may have a role in facilitating successful retirement adjustment, a view also supported by qualitative findings highlighting the role of LTPA in providing structure and meaningful daily content in the lives of the recently retired (Barnett et al. 2012; Beck et al. 2010; Kosteli et al. 2016; Rai et al. 2020). However, it has also been suggested that the changes in LTPA across retirement transition might actually be partly dependent on (un)successful adjustment to changes in daily life circumstances brought by retirement (Rai et al. 2020). Furthermore, it is possible that the observed associations between life satisfaction and LTPA are partly driven by factors associated with both, such as health or socioeconomical status.

Accordingly, a recent systematic review concluded that while LTPA tends to associate positively with life satisfaction and other retirement adjustment indicators, the nature of this association remains unclear (Sharifi et al. 2023). For example, positive cross-sectional associations between levels of LTPA and life satisfaction among retirement-aged adults have been consistently reported (Conde-Sala et al. 2017; Kuvaja-Köllner et al. 2013; Lee and Hung 2011), but only a few studies have examined associations between concurrent changes in LTPA and life satisfaction across retirement transition. In one Australian study, reallocating time previously spent in work to LTPA was not observed to be associated with changes in life satisfaction across retirement transition, albeit an association with reduced negative affectivity was observed (Olds et al. 2018). Another Australian study observed that retirees who retrospectively reported increasing their levels of physical activity across retirement also tended to report higher levels of life satisfaction after retirement (Noone et al. 2013). However, as changes in life satisfaction were not examined, the observed association could simply mean that those with high life satisfaction before retirement may be more likely to increase their LTPA across retirement transition.

The current study hypothesized that changes in life satisfaction would be associated with concurrent changes in LTPA among Finnish public sector workers transitioning to age-based retirement. Given that ample evidence suggests levels of life satisfaction and LTPA to be positively associated, the associations between co-occurring changes were examined while taking into account the level of life satisfaction before retirement. More specifically, it was hypothesized that increasing or decreasing levels of life satisfaction would be associated with, accordingly, more or less favorable changes in LTPA when compared to those with maintained levels of life satisfaction across retirement transition.

## Materials and methods

### Study design and participants

The current study is based on the Finnish Retirement and Aging (FIREA) longitudinal cohort study established in 2013. The FIREA study design and recruitment is described in detail elsewhere (Stenholm et al. 2023). Briefly, the eligible study population included Finnish public sector employees working in any town in Southwest Finland, or in any of the a priori selected nine towns or five hospital districts across Finland in 2012, and with an estimated retirement date between 2014 and 2019 (*n* = 10,629). The participants were invited to participate in the FIREA study by sending them a questionnaire 18 months prior to their estimated statutory retirement date, which was drawn from the register of the institute for public sector pensions (Keva). Thereafter the follow-up study questionnaires were sent annually with one-year interval to the participants with the aim to obtain both pre- and post-retirement measurements. The FIREA study was approved by the ethics committee of the Hospital District of Southwest Finland (84/1801/2014), and informed consent was obtained from all participants.

**Fig. 1:**
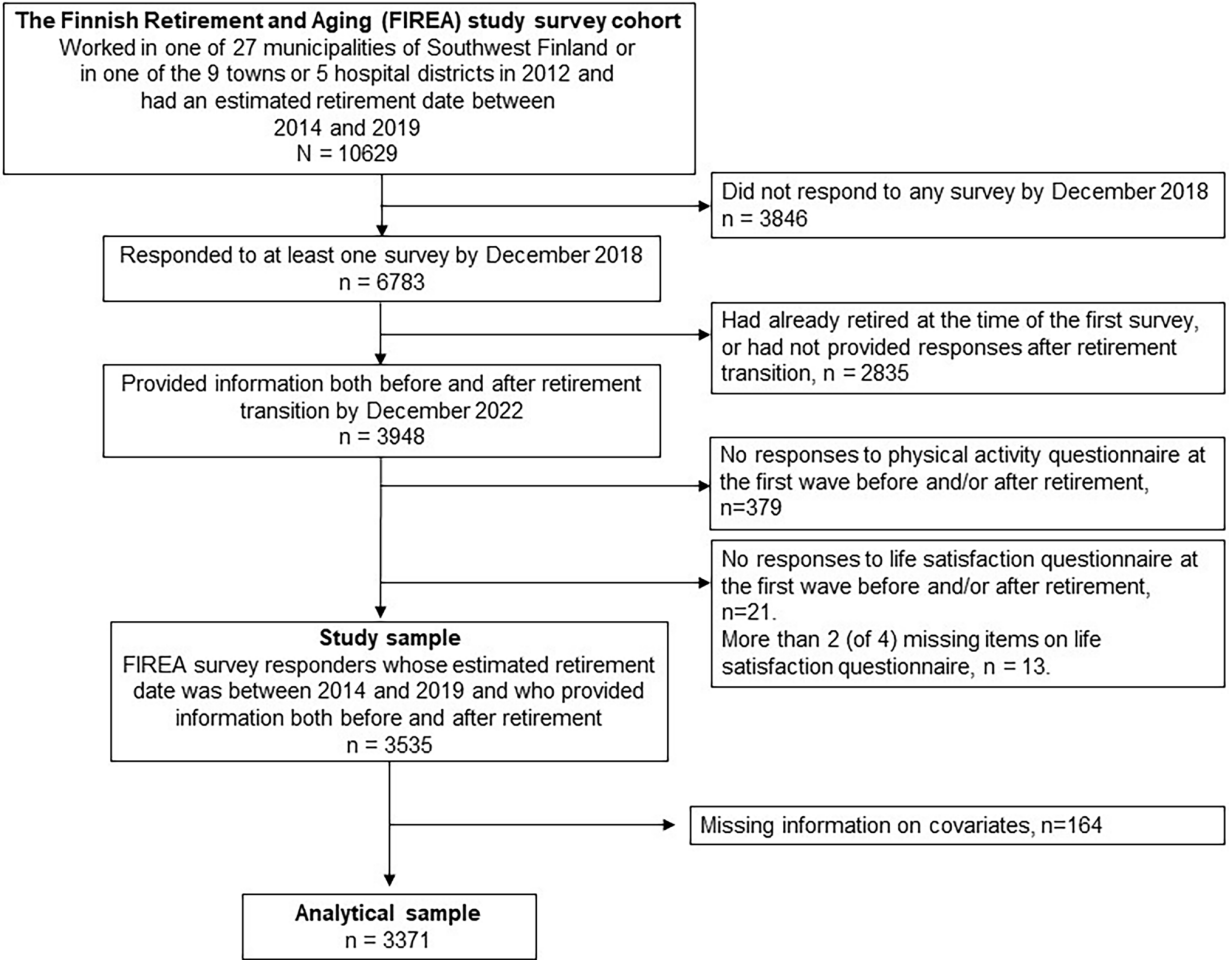
Flowchart of the selection of the study sample

The survey data were centered on retirement transition based on self-reported actual retirement date inquired during each phase of the data collection. For the present study, one data collection wave preceding (wave −1) and three waves following (wave +1, wave +2, wave +3) retirement transition were included, with retirement transition defined as the time period between wave −1 and wave +1. Of the 10,269 invited to participate in the FIREA survey cohort, altogether 6783 participants responded to at least one survey by December 2018, of which 2835 were excluded due to being already retired by their first response, or not providing any survey responses after retirement transition. Thus, the FIREA study cohort mainly included participants reaching their statutory retirement age. By the end of year 2022, 3948 FIREA participants had provided at least one response to the study questionnaire both before and after retirement, of which 3548 participants had provided information on both LTPA and life satisfaction at both wave −1 and wave +1 required for inclusion in the present study. The sample selection for the present study is further illustrated in Fig. 1.

### Measurement of physical activity

*Leisure-time physical activity (LTPA)* was assessed with four items considering the average weekly duration of time spent in physical activities equivalent of a) light walking, b) brisk walking, c) jogging, and d) running in leisure-time or commuting during the past year (Kujala et al. 1998). There were five response options for each intensity class and the following values were used for calculating the weekly hours spent in physical activity: “Not at all” (0h), “Less than 30 min” (0.25 h), “Approximately one hour” (0.75h), “2–3 h” (2.5 h), and “Four hours or more” (5 h) in line with previous uses of the scale (Stenholm et al. 2016). Time accumulated at each intensity level was multiplied with a given metabolic equivalent task (MET) multiplier (3.5 for light walking, 5 for brisk walking, 8 for jogging and 11 for running) (Ainsworth et al. 2011) and summed to represent intensity-weighted total amount of weekly LTPA expressed as METh/week. Although the questionnaire wording included commuting, the physical activity outcome will be referred to as LTPA for brevity, and to highlight the fact that it does not consider occupational physical activity. Altogether 3548 participants provided information on LTPA at waves −1 and +1, 2826 at wave +2, and 1440 at wave +3.

### Measurement of life satisfaction

*Life Satisfaction* was measured with a four-item scale, including assessments of happiness, interestingness, and easiness of life, as well as one item on perceived loneliness (Koivumaa-Honkanen 2000). The scale was developed from quality of life studies for a measure of welfare in the Nordic Countries (Allardt 1976), and it has since been used in several studies to predict a range of health and behavioral outcomes, including physical activity (Koivumaa-Honkanen 2000; Stenlund et al. 2021a, c). The questionnaire stem was “Do you feel that your life is currently…”, and the response options were scored and summed in line with previous procedures (i.e., 1 = ”very boring/unhappy/hard/lonely”; 2 = “boring/unhappy/hard/lonely”; 3 = ”cannot say”; 4 = fairly interesting/happy/easy”; 5 = “very interesting/happy/easy/not at all lonely”; sum score range 4–20) (Koivumaa-Honkanen 2000). If one of the four items was missing, the missing item was replaced by the average of the existing three items in order to calculate the sum score (calculated value for *n* = 34 at wave − 1 and *n* = 28 at wave + 1) in line with previous procedures (Stenlund et al., 2021b). If more than one of the four items was missing at wave −1 (*n* = 7), wave +1 (*n* = 5), or both (*n* = 1), the participants were excluded from the analysis. Cronbach alpha for the life satisfaction scale was *α* = 0.77 at wave −1 and *α* = 0.78 at wave +1. The level of life satisfaction was categorized separately at wave −1 and wave +1 into low (sum score 4–12), intermediate (13–17) and high life satisfaction (18–20) based on previously used cut-points originally drawn from ± 1 standard deviation limits from a large study with a nationwide sample representative of the Finnish population aged 18–64 years (Koivumaa-Honkanen 2000). Finally, nine possible groups (3 × 3) were created based on the categorized levels of life satisfaction at wave −1 and wave +1 to reflect changes in life satisfaction across retirement transition. As the changes from high to low (*n* = 4) and low to high levels of life satisfaction (*n* = 25) were rare, the respective groups were combined with high to intermediate and low to intermediate groups. Thus, seven life satisfaction change groups across retirement transition were used in the analyses: Stable Low, Low-Increasing, Intermediate-Decreasing, Stable Intermediate, Intermediate-Increasing, High-Decreasing, and Stable High.

### Covariates

Age, gender, and last known occupational title preceding retirement were obtained from the register data of the institute for public sector pensions in Finland (Keva). The occupational titles were classified using the International Standard Classification of Occupations (ISCO) and categorized as follows: “High” including managers and professional, such as teachers and doctors (ISCO classes 1 and 2), “Intermediate” including associate professionals, such as registered nurses, and clerical workers (ISCO classes 3 and 4), and “Low” including manual and service workers, such as market sales workers, cooks, and maintenance workers (ISCO classes 5 to 9). Marital status, self-rated health, mobility limitations, and body mass index (BMI) were obtained from wave −1 and wave +1, or if missing, from wave −2 and wave +2, respectively. Marital status was dichotomized into married/cohabiting and never married/divorced/separated/widowed. Self-rated health was dichotomized into good (good/rather good) and suboptimal (average/rather poor/poor). Mobility limitations were assessed with a single-item assessing the extent to which current health status limits walking 2 km and the responses were dichotomized into no (not limiting) and yes (limits some/a lot). BMI was calculated based on the self-reported weight and height (kg/m^2^). Age, gender, occupational background, marital status, and health status indicators were included as covariates as they have been previously linked with changes in both LTPA and life satisfaction.

### Statistical analyses

Participant characteristics were described using frequencies and percentages for categorical and mean and SD for continuous variables. Overall changes in LTPA and life satisfaction across retirement transition were examined with paired samples t-tests. To illustrate changes in LTPA across life satisfaction change groups, means and 95% confidence intervals were calculated for the levels of METh/week of LTPA from wave −1 to wave +3.

For the main analysis, retirement transition was defined as the time period between wave −1 and wave +1, representing time points before and after retirement transition. The main analyses were restricted to the participants providing full information on covariates. Linear models with generalized estimating equations (GEE) were used to examine differences in changes in LTPA based on concurrent changes in life satisfaction across retirement transition. The unadjusted model included time as a two-level categorical within-factor, life satisfaction change group as a seven-level categorical between-factor, and their interaction term. METh/week was a continuous dependent variable. Contrast statements were used to compare for mean differences in the mean changes in METh/week between different life satisfaction change groups. The comparisons of interest were conducted within each level of life satisfaction before retirement and against the group representing no change, i.e., Stable Low vs. Low-Increasing, Stable Intermediate vs. Intermediate-Decreasing, Stable Intermediate versus Intermediate-Increasing, and Stable High vs. High-Decreasing. The models were adjusted for age, gender, occupational background, and marital status (Model 1), and further with self-rated health, mobility limitation, and BMI (Model 2). Gender and occupational background were treated as fixed covariates with main and interaction effects with time included in the models. Age, marital status, self-rated health, mobility limitations, and BMI were included as time-varying covariates. All analyses were conducted with SAS (version 9.4; SAS Institute Inc, Cary, NC, USA).

Additional analyses were conducted with METh/week categorized into 1 = < 14METh/week, 2 = 14-30METh/week, 3 = > 30METh/week, in line with previous uses of the scale (Leskinen et al. 2018). Although used in several previous studies, the physical activity scale has not been formally validated and thus may be subject to unknown amount of bias and inaccuracy. Consequently, cumulative logistic regression was used to examine the cumulative odds ratio for higher activity category at wave +1 when compared to wave −1 across life satisfaction change groups.

Further sensitivity analyses were also conducted. The main analyses were replicated using sample-specific cut-points for classifying the levels of life satisfaction as the sample of the current study consisted solely of retirement-aged individuals. For sensitivity analyses, mean and SD from wave −1 was used for classifying the levels of life satisfaction at both wave −1 and wave +1. Those with a life satisfaction sum score more than 1 SD below the mean were classified as having low life satisfaction (sum score < 14), those with a sum score within 1 SD from the mean were classified as having intermediate life satisfaction (sum score 14–19), and those with a sum score more than 1 SD above the mean were classified as having high satisfaction (sum score = 20). In addition, the main analyses were further repeated while a) using multiple imputation by chained equations for missing information on covariates (Azur et al. 2011), and b) among a subgroup of 2754 participants not reporting mobility limitations either before or after retirement.

## Results

The characteristics of the 3535 participants providing information on both life satisfaction and LTPA are presented in Table 1. At wave −1, the participants were on average 63.4 (SD 1.4) years old (range 58.0 to 68.0 years old), mainly women (83%), married/cohabiting (71%), representing different occupational backgrounds (34% High, 31% Intermediate, 35% Low), reporting mainly good or rather good self-rated health (76%) and no mobility limitation (87%), with the average BMI being 26.8 (SD 4.5) kg/m^2^. Before retirement, 8.4% of the participants were classified as having low, 56.4% intermediate, and 35.2% high levels of life satisfaction. Participants with low life satisfaction tended to report worse self-rated health, more mobility limitations, had higher body mass index, were less likely to be married/cohabiting, and had the lowest levels of LTPA. Participants with high life satisfaction before retirement, in turn, were more likely to have high occupational background, report better self-rated health and less mobility limitations, were more likely to be married/cohabiting, and had the highest levels of LTPA (Online Supplementary Table S1). In terms of categorized levels of METh/week before retirement, suboptimal self-rated health and mobility limitations were most frequently reported among participants with < 14METh/week who also reported highest BMI and lowest life satisfaction before retirement (Online Supplementary Table S2).

**Table 1:** Participants characteristics before and after retirement among 3535 participants providing information on life satisfaction and LTPA

Characteristics	Last wave^a^ before retirement		First wave^a^ after retirement	
	*n*		*n*	
**Age, Years**, **mean (SD)**	3535	63.4 (1.4)	3535	64.4 (1.4)
**Sex, % (n)**	3535		3535	
Women		83.2% (2940)		83.2% (2940)
Men		16.8% (595)		16.8% (595)
**Occupational background, % (n)**	3507		3507	
High		34.0% (1194)		34.0% (1194)
Intermediate		31.0% (1087)		31.0% (1087)
Low		35.0% (1226)		35.0% (1226)
Missing	28		28	
**Partner status, % (n)**	3492		3502	
Married/cohabiting		71.2% (2486)		71.4% (2501)
Not married/divorced/widowed		28.8% (1006)		28.6% (1001)
Missing	43		33	
**Self-rated Health, % (n)**	3533		3531	
Good		76.1% (2684)		80.6% (2845)
Suboptimal		23.9% (844)		19.4% (686)
Missing	2		4	
**Mobility limitations, % (n)**	3524		3523	
No		86.6% (3053)		87.1% (3069)
Yes		13.4% (471)		12.9% (454)
Missing	11		12	
**Body mass index, kg/m2, mean (SD)**	3512	26.8 (4.5)	3517	26.8 (4.5)
Missing	23		28	

^a^On average 0.5 years before/after retirement or, if missing, 1.5 years before/after retirement

The average level of LTPA increased from 23.8 (SD 19.8) METh/week at wave −1 to 26.0 (SD 20.50) METh/week at wave +1, the mean change being 2.16 METh/week (95% CI 1.53 to 2.79). Similarly, the average life satisfaction increased from 16.6 (SD 2.6) to 17.0 (SD 2.5) during retirement transition, the mean change being 0.39 (95% CI 0.32 to 0.46). In terms of changes in the levels of life satisfaction, two thirds of participants maintained their level of life satisfaction, while this level increased in one fifth and decreased in one tenth of the participants from wave − 1 to wave + 1.

**Fig. 2:**
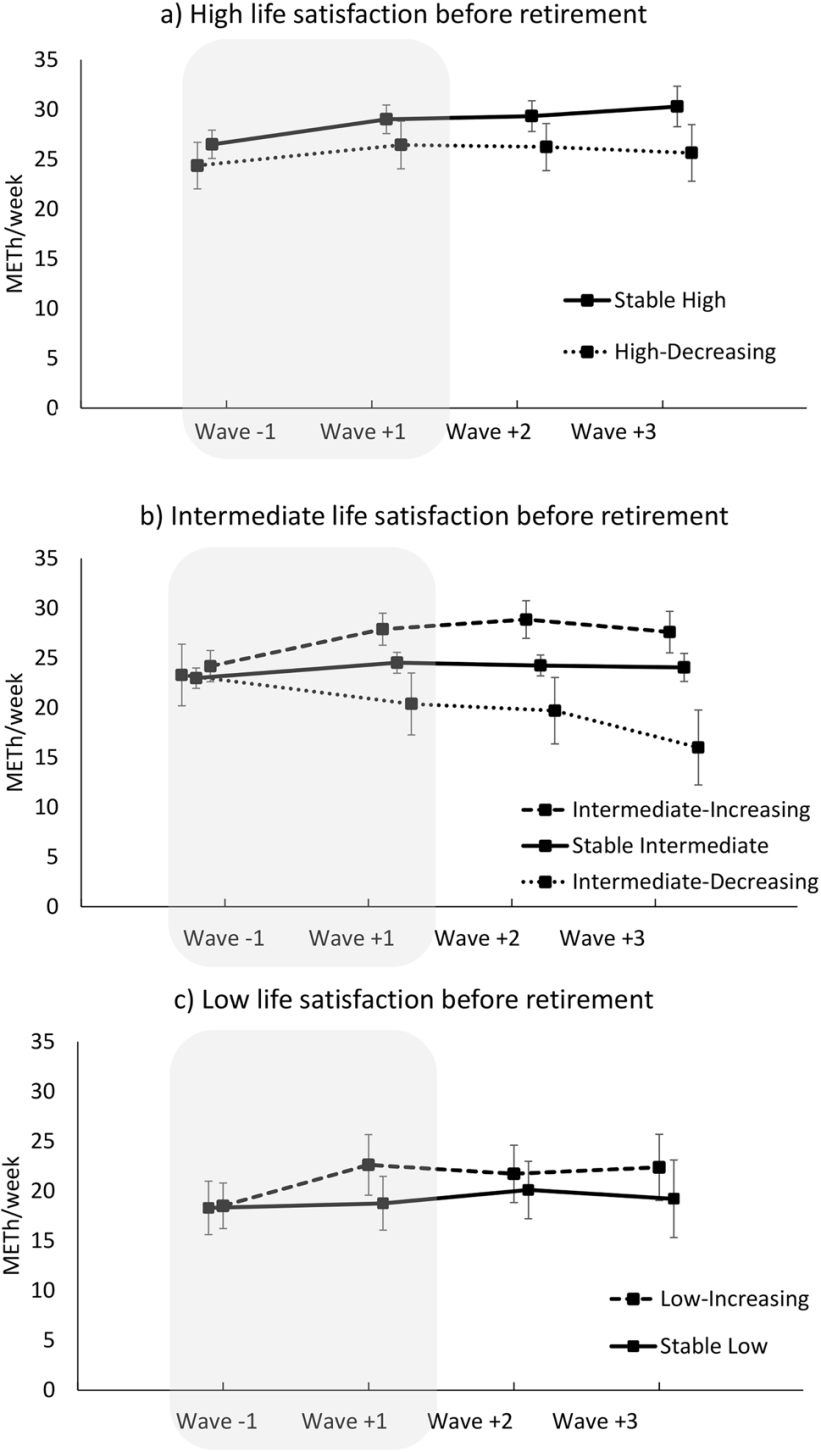
Mean levels of self-reported leisure-time physical activity (METh/week) with 95% confidence intervals across life satisfaction change groups among participants with **A** High, **B** Intermediate, and **C** Low life satisfaction before retirement. The shaded area represents retirement transition period with one year between the study waves; Wave −1, *n* = 3535, Wave + 1, *n* = 3535, Wave + 2, *n* = 2816, Wave + 3, *n* = 1434

The observed levels of LTPA from wave −1 to wave +3 by the life satisfaction change groups are illustrated in Fig. 2. Overall, the average levels of LTPA tended to increase across retirement transition from wave −1 to wave +1 and decline during post-retirement. However, among those with Intermediate-Decreasing life satisfaction across retirement transition, a consistent decline in the levels of LTPA was observed. Among those with Low-Increasing life satisfaction, a transient increase in the levels of LTPA was observed, while no changes in LTPA were observed among those with Stable Low life satisfaction.

The main analyses were restricted to 3371 participants providing full information on covariates at wave −1 and wave +1 with participant characteristics presented in Table 2. Overall, there was a statistically significant reduction in the proportion of participants reporting suboptimal self-rated health from 23.8% before retirement to 19.2% after retirement. No differences in other covariates across retirement transition were observed, apart from a one-year increase in the average age attributable to the annually spaced measurements. There were some differences in covariate distributions across life satisfaction change groups observed but these were mainly associated with the level of life satisfaction before retirement. However, highest declines in the prevalence of suboptimal self-rated health were observed among Low-Increasing and Intermediate-Increasing life satisfaction, whereas the proportion of participants reporting mobility limitations was observed to increase only among Intermediate-Decreasing life satisfaction from wave −1 to wave +1 (Online Supplementary Table S3).

**Table 2:** Participant characteristics before and after retirement among 3371 participants providing full information on covariates

Characteristics	Last wave^a^ before retirement	First wave^a^ after retirement	*p*-value
	*n* = 3371	*n* = 3371	
**Age, Years, mean (SD)**	63.4 (1.4)	64.4 (1.4)	<.001^b^
**Sex, % (n)**			
Women	82.7% (2789)	82.7% (2789)	n/a
Men	17.3% (582)	17.3% (582)	
**Occupational background, % (n)**			n/a
High	34.4% (1160)	34.4% (1160)	
Intermediate	31.1% (1047)	31.1% (1047)	
Low	34.5% (1164)	34.5% (1164)	
**Partner status, % (n)**			.823^c^
Married/cohabiting	71.4% (2408)	71.4% (2406)	
Not married/divorced/widowed	28.6% (963)	28.6% (965)	
**Self-rated health, % (n)**			<.001^c^
Good	76.2% (2567)	80.8% (2722)	
Suboptimal	23.8% (804)	19.2% (649)	
**Mobility limitations, % (n)**			.557^c^
No	86.7% (2924)	87.1% (2935)	
Yes	13.3% (447)	12.9% (436)	
**Body mass index, kg/m2, mean (SD)**	26.8 (4.5)	26.9 (4.6)	.158^b^

^a^on average 0.5 years before/after retirement or, if missing, 1.5 years before/after retirement

^b^Paired samples t test; ^c^McNemar’s Chi-square test

Table 3 presents the changes in LTPA across the retirement transition period across life satisfaction change groups. In the unadjusted model, changes in LTPA were associated with concurrent changes in life satisfaction (interaction time*life satisfaction change group, *p* = 0.004) with the inclusion of sociodemographic covariates having minimal impact on the observed results. Furthermore, statistically significant differences in changes in LTPA were observed across life satisfaction change groups while adjusting for both sociodemographic and health-related covariates (Model 2, interaction time*life satisfaction change group, *p* =. 018). Low-Increasing life satisfaction was associated with a concurrent increase in LTPA across the retirement transition period, when compared to Stable Low life satisfaction among whom no changes were observed (Mean difference in mean change: 4.16 METh/week, 95% CI 0.85 to 7.47). Similarly, Intermediate-Increasing life satisfaction was associated with a higher increase in METh/week across retirement transition when compared to the Stable Intermediate life satisfaction (Mean difference in mean change: 1.96 METh/week, 95% CI 0.03 to 3.88). Intermediate-Decreasing life satisfaction was, in turn, associated with less favorable changes in LTPA across the retirement transition period when compared to Stable Intermediate life satisfaction (Mean difference in mean change: −3.79 METh/week, 95% CI −7.62 to 0.04), albeit this difference was not statistically significant after adjusting for health-related covariates. No statistically significant differences in the changes of LTPA were observed between those with High-Decreasing and Stable High life satisfaction.

**Table 3:** Model-based mean changes in leisure-time physical activity across retirement transition in life satisfaction change groups with 95% confidence intervals (95% CI)

Life satisfaction across retirement transition	Stable Low	Low-increasing	Intermediate-Decreasing	Stable Intermediate	Intermediate-Increasing	High-decreasing	Stable High	p-value^a^
LTPA (METh/week)	*n* = 133	*n* = 151	*n* = 83	*n* = 1271	*n* = 553	*n* = 302	*n* = 878	Time X group
**Unadjusted model**	**Mean (95% CI)**							*p* =.004
Mean change from wave −1 to wave + 1	0.73 (−1.08 to 2.54)	5.12 (2.36 to 7.88)	−2.74 (−6.33 to 0.86)	1.55 (0.52 to 2.57)	3.81 (2.20 to 5.41)	1.91 (−0.35 to 4.18)	2.62 (1.24 to 4.00)	
Mean difference in mean change	ref	4.39 (1.09 to 7.69)	−4.28 (−8.02 to −0.55)	ref	2.26 (0.36 to 4.16)	−0.71 (−3.36 to 1.94)	ref	
**Model 1**^**b**^	**Mean (95% CI)**							*p* =.004
Mean change from wave −1 to wave + 1	0.30 (−1.65 to 2.24)	4.78 (1.95 to 7.61)	−3.09 (−6.74 to 0.57)	1.19 (0.00 to 2.38)	3.43 (1.64 to 5.22)	1.58 (−0.85 to 4.02)	2.22 (0.62 to 3.82)	
Mean difference in mean change	ref	4.48 (1.19 to 7.77)	−4.28 (−8.01 to −0.55)	ref	2.24 (0.33 to 4.15)	−0.64 (−3.28 to 2.00)	ref	
**Model 2**^**c**^	**Mean (95% CI)**							*p* =.018
Mean change from wave −1 to wave + 1	0.28 (−1.80 to 2.36)	4.44 (1.67 to 7.20)	−2.44 (−6.20 to 1.31)	1.35 (0.17 to 2.53)	3.31 (1.50 to 5.11)	1.89 (−0.55 to 4.32)	2.42 (0.83 to 4.01)	
Mean difference in mean change	ref	4.16 (0.85 to 7.47)	−3.79 (− 7.62 to 0.04)	ref	1.96 (0.03 to 3.88)	−0.54 (−3.19 to 2.11)	ref	

^a^p-value is for the interaction term time X life satisfaction change group

^b^Adjusted for age, gender, occupational background, and marital status

^c^Adjusted for age, gender, occupational background, marital status, self-rated health, mobility limitations, and body mass index

In the cumulative logistic regression on categorized METh/week outcomes, the observed effect sizes for between-group contrasts were in line with the results attained from the main analyses. Highest increases in cumulative odds for higher activity were observed among Low-Increasing and Intermediate-Increasing life satisfaction, while no differences between High-Decreasing and Stable High life satisfaction were observed (Online Supplementary Tables S4 and S5). In addition, the overall results remained mainly similar with comparable effect sizes for between-group contrasts while using sample-specific cut-points for classifying the levels of life satisfaction (Online Supplementary Table S6). However, in contrast to main findings, there was an indication of a difference in changes in METh/week between High-Decreasing and Stable High life satisfaction. Yet, the use of sample-specific cut-points had a large impact on classified frequencies with high life satisfaction requiring, in effect, maximum score on the scale. The main findings were further replicated by using five imputed data sets for missing covariates (Model 2 interaction time*life satisfaction change group, average *p* = 0.020). After adjusting for sociodemographic and health-related covariates, the mean estimates for between-group contrasts were similar to those attained from the main analytical models with the addition of the contrast between Stable Intermediate and Intermediate-Decreasing being statistically significant with imputed data (mean difference −3.94 METh/week, 95% CI −7.60 to −0.28) (Online Supplementary Table S7). In addition, the effect sizes of the between-group comparisons were also similar to main findings when restricting the analysis to the 2754 participants not reporting mobility limitations either before or after retirement (Online Supplementary Table S8).

## Discussion

Current findings suggest that changes in life satisfaction across retirement transition are likely to be associated with concurrent changes in LTPA, especially among those with lower and intermediate levels of life satisfaction before retirement. Life satisfaction increasing from low and intermediate levels across retirement transition was found to be associated with highest increases in LTPA. Life satisfaction declining to low level, in turn, tended to be associated with decreasing LTPA. In addition, consistently low life satisfaction was associated with lowest levels of LTPA both before and after retirement. No associations between changes in life satisfaction and concurrent changes in LTPA were observed among those with high life satisfaction preceding retirement.

The observed changes in LTPA across retirement ranged approximately from a decline of 2.5 METh/week to an increase of 4.5 METh/week. These can be translated roughly to 30–54 min of brisk walking per week, respectively, using the MET-reference values (Ainsworth et al. 2011). The increases within this magnitude may seem relatively small when compared to the number of weekly hours freed from previous work-related responsibilities. Yet, any decline or lack of increase should be considered alarming for the same reason. Furthermore, if maintained, even relatively minor changes across retirement transition may have long-lasting consequences, given the importance of regular physical activity for healthy aging (Bangsbo et al. 2019).

The repeated data centered on retirement date with one-year measurement intervals allowed for focusing on the retirement transition period, a possibility missing from the majority of previous studies (Barbosa et al. 2016; Sharifi et al. 2023). Overall, our findings add to previous observations that the levels of both LTPA and life satisfaction tend to increase following retirement (Hansson et al. 2018; Prakash et al. 2022; Xue et al. 2020). More importantly, our observations indicate that the changes in life satisfaction across retirement transition are associated with concurrent changes in LTPA. Thus, our findings are in agreement with a previous qualitative study identifying the adoption of either a gain or loss approach to retirement as an important factor in influencing LTPA engagement following retirement (Rai et al. 2020).

However, there were clear differences observed in self-rated health and mobility limitations across categories of life satisfaction in accordance with previous studies (Hansson et al. 2018; Heybroek et al. 2015; Pinquart and Schindler 2007; Prakash et al. 2022). Health and mobility-related factors are also likely to be associated with changes in LTPA across retirement (Barnett et al. 2012; Beck et al. 2010; Jones et al. 2018a; Rai et al. 2020; Stenholm et al. 2016). Accordingly, greatest increases in both self-rated health and METh/week were observed among participants with increasing life satisfaction. Participants with Stable Low life satisfaction, in turn, reported highest levels of suboptimal self-rated health and mobility limitations and lowest levels of METh/week both before and after retirement. Furthermore, Intermediate-Decreasing life satisfaction was the only class among whom mobility limitations increased, while also being the only class with an observed negative mean change in METh/week. There were no differences in changes in LTPA observed between participants with Stable High and High-Decreasing life satisfaction, among whom both poor health and mobility limitations were most infrequent. It is then possible that there are changes in health-related factors co-occurring with retirement transition, which serve to facilitate or hinder both LTPA and life satisfaction. However, the sensitivity analysis conducted among participants never reporting mobility limitations suggests that the observed association between life satisfaction and LTPA across retirement transition is unlikely to be fully due to co-occurring changes in physical health.

The impact of retirement on the levels of LTPA is likely to be influenced by the perceived nature and desirability of the changes brought about by retirement. Previous studies suggest that increases in life satisfaction are mainly driven by improvements in daily life’s circumstances following retirement, for example, through alleviation of demanding or stressful working conditions (Wang 2007), increased autonomy (Hansson et al. 2018), or increased overall easiness of living (Prakash et al. 2022). These changes are likely to free individual’s resources, particularly in terms of time and energy, which is then suggested to facilitate LTPA engagement following retirement (McDonald et al. 2015). It has also been argued that the presence of work-related stressors may produce self-regulatory fatigue and reduce global self-determined motivation, both of which may be required for LTPA engagement (Häusser and Mojzisch 2017). Thus, the alleviation of work-related stressors would be expected to result in increases in both life satisfaction and LTPA after retirement. This might be the case particularly among those with poor health, low fitness, or mobility limitations among whom the negative impacts of work-related demands and stressors, either physical or psychological, might be felt more strongly. Additionally, higher occupational level is likely to be associated with lower work-related physical demands and higher control over the content of one’s work, a central factor in work stress theories (Häusser and Mojzisch 2017), as well as with better financial resources shown to be positively associated with changes in life satisfaction (Hansson et al. 2018). Therefore, the extent of changes in resource and energy availability assumed to foster LTPA engagement at retirement might differ based on the health and socioeconomic status of the individual. This could also partly explain our findings, given that those with high life satisfaction before retirement were most likely to have not only better health, but also high occupational background.

Finally, the levels of life satisfaction and LTPA tend to be positively associated (Kuykendall et al. 2015; Wiese et al. 2018), with previously established LTPA habits consistently identified as a central facilitator for adopting and maintaining LTPA following retirement (Beck et al. 2010; Kosteli et al. 2016; Rai et al. 2020; Ter Hoeve et al. 2020). Participants with high levels of life satisfaction before retirement may then both be more likely to increase LTPA and be less likely to experience major changes in life satisfaction across retirement transition. This may also partly explain why previous studies have found associations between LTPA and levels (Conde-Sala et al. 2017; Kuvaja-Köllner et al. 2013; Lee and Hung 2011; Noone et al. 2013), but not changes (Olds et al. 2018) of life satisfaction among retirement-aged individuals. Nevertheless, qualitative findings suggest that those with established LTPA habits before retirement may be more likely to use LTPA for providing structure and meaningful daily content in their post-retirement lives (Beck et al. 2010; Kosteli et al. 2016; McDonald et al. 2015; Rai et al. 2020). Therefore, further research on the directionality of the association and its potential dependency on the levels of LTPA before retirement is warranted.

### Strengths and limitations

The current study included repeated measurements from a large cohort centered on actual retirement transition time with 1-year measurement intervals. Repeated measurements allow for focusing on intra-individual changes, while reducing the impact of unmeasured time-invariant confounders, and taking into consideration time-varying confounders. For example, health and mobility status are likely to be strongly associated with both LTPA and life satisfaction. Accordingly, the inclusion of health-related factors slightly attenuated some of the observed differences. Yet, the effect sizes for between-group contrasts remained similar in the presence of time-varying health-related covariates with the main findings further replicated while using cumulative logistic regression on categorized LTPA outcomes, multiple imputation, and including only participants never reporting mobility limitations.

Naturally there are also number of limitations that should be addressed. First, both life satisfaction and LTPA measures were self-reported, thus subject to a number of biases, namely recall, social desirability bias, and common method bias. However, while these biases are likely to have implications for the reported levels, their impact on changes is less clear, assuming that the reporting style remains similar across time points. Furthermore, self-reported measures are well-suited for examining changes occurring in physical activity across retirement transition as they avoid problems associated with distinguishing between physical activity accumulated during work and leisure-time preceding retirement. Yet, the self-report instrument included both leisure-time and commuting-related physical activity. Given that commuting-related time is by definition restricted to pre-retirement measurements, it is possible that the changes observed in LTPA would have been more pronounced had the questionnaire been focused solely on leisure time. On a similar note, it is also possible that the observed declines or lack of changes in LTPA are partly due to loss of commuting-related physical activity rather than declining or not changing leisure-time physical activity levels.

Second, the proportion of participants classified as having low life satisfaction, either before or after retirement, was low. This may indicate that those with low levels of life satisfaction tend to be less inclined to participate in studies in general. However, the current study was also focused on participants reaching age-based retirement, among whom the levels of life satisfaction may be higher when to compared to similarly aged people retiring/not working for other than age-based reasons. This was also suggested by the use of sample-specific 1 SD cut-points requiring maximum score on the scale to be classified as having high life satisfaction. Lower average levels of life satisfaction have been observed among adults of similar age in earlier studies using the same life satisfaction measure in more representative samples of the Finnish population (Koivumaa-Honkanen 2000; Stenlund et al., 2021b). In the current sample, low levels of life satisfaction were also less and high levels more frequently reported (Stenlund et al. 2021c). Suboptimal self-rated health was also clearly less frequent when compared to participants of similar age included in European samples more representative of the population, including Finland (Bardage et al. 2005). This is likely to reflect a form of healthy worker effect as people able to work until their statutory retirement date might be more likely to have better health and well-being, and may, consequently, also be more likely to engage in LTPA when compared to similarly aged population at large.

Third, the number of men in the sample was small, although the gender distribution of the sample is representative of public sector workers in Finland of whom 78% are women (Statistics Finland 2016). Although previous studies have not observed gender-based differences in changes in LTPA across retirement, there may be between-gender variability in the forms of physical activity engagement, types and preferences. For example, men may be more likely to engage in LTPA as a hobby providing personal challenges, whereas women may find instrumental purposes of LTPA, such as fostering social interaction, more important (Barnett et al. 2012; Beck et al. 2010). This may have implications for both the expected changes in LTPA across retirement and for their potential sensitivity to co-occurring changes in life satisfaction. In addition, high occupational background was overrepresented in the sample (35%) when compared to the non-participants of the FIREA survey (26%) (Stenholm et al. 2023), although different occupational backgrounds were evenly represented in the sample. However, high occupational background was more common among men (49.8%) than women (31.2%). While this may suggest stronger self-selection among men, it is also likely to be partly associated with imbalanced occupational distributions among public sector workers in Finland. However, due to low number men in the sample, no meaningful subgroup analyses based on gender could be conducted, which limits the generalizability of the current findings. Finally, there are differences in retirement schemes and pension levels between countries, as well as between different occupations within countries, which may have an impact on the experiences associated with retirement. However, these differences may be more likely to have implications for changes in life satisfaction rather than for the association between concurrent changes life satisfaction and LTPA across retirement transition. Consequently, the findings of the current study can be mainly generalized to women transitioning to age-based retirement in Nordic, or similar, contexts.

## Conclusion

The results of the current study propose that changes in life satisfaction across retirement transition are associated with concurrent changes in LTPA, particularly among those with lower or intermediate levels of life satisfaction before retirement. These findings suggest that supporting life satisfaction during retirement transition could foster greater engagement in physical activity, offering potential avenues for public health interventions. For example, health professionals working with retirees should consider monitoring life satisfaction during the retirement transition and offering interventions aimed at improving well-being as a means to boost physical activity levels. Overall, the current findings highlight the need to consider the broader experience of retirement when aiming to promote physical activity among recent retirees, either at individual or population level.

## Supplementary Information

Below is the link to the electronic supplementary material.

Supplementary file1 (PDF 283 KB)

## Data Availability

Anonymized partial datasets of the FIREA study are available by application with bona fide researchers with an established scientific record and bona fide organizations. In case of data requests, please contact the principal investigator Sari Stenholm, sari.stenholm@utu.fi.
