# Supplementary material for: Changes in life satisfaction and leisure-time physical activity across retirement transition: the FIREA cohort study
Source: Eur J Ageing. 2025 Jun 17;22(1):29. doi: 10.1007/s10433-025-00865-x (PMC12173991; doi:10.1007/s10433-025-00865-x)
Supplement: Supplementary file 1 — Supplementary file1 (PDF 283 KB) [file 10433_2025_865_MOESM1_ESM.pdf]

**Supplementary Table S1.** Participant characteristics across levels of life satisfaction before retirement among the 3535 participants providing information on both life satisfaction and leisure-time physical activity before and after retirement.

| | Level of life satisfaction before retirement | | | |
|---|---|---|---|---|
| **Total,** **n=3535** | **Low** | **Intermediate** | **High** | **p-value** |
| | | | | |
| n (%), n=3535 | 301 (8.5%) | 2008 (56.8%) | 1226 (34.7%) | |
| | | | | |
| Age, years, mean (SD), n=3535 | 63.5 (1.4) | 63.3 (1.4)[a] | 63.5 (1.4)[a] | .001 |
| | | | | |
| Women, n (%), n=3535 | 258 (85.7%) | 1654 (82.4%) | 1028 (83.9%) | .258 |
| | | | | |
| Occupational background, n (%), n=3507 | | | | <.001 |
| High | 86 (28.7%)[a] | 627 (31.6%)[b] | 481 (39.4%)[a,b] | |
| Intermediate | 109 (36.3%) | 646 (32.5%) | 332 (27.2%) | |
| Low | 105 (35.0%) | 713 (35.9%) | 408 (33.4%) | |
| | | | | |
| Married/cohabiting, n (%), n=3492 | 149 (50.5%)[a,c] | 1400 (70.6%)[a,b] | 937 (77.3%)[b,c] | <.001 |
| | | | | |
| Suboptimal self-rated health, n (%), n=3533 | 156 (51.8%)[a,c] | 543 (27.1%)[a,b] | 146 (11.9%)[b,c] | <.001 |
| | | | | |
| Mobility limitations, n (%), n=3524 | 78 (26.0%)[a,c] | 283 (14.2%)[a,b] | 110 (9.0%)[b,c] | <.001 |
| | | | | |
| BMI, kg/m$^2$, mean (SD), n=3512 | 27.7 (5.3)[a,b] | 26.8 (4.3)[a] | 26.7 (4.6)[b] | .004 |
| | | | | |
| METh/week at wave -1, mean (SD), n=3535 | 18.4 (15.5)[a,c] | 23.3 (19.0)[a,b] | 26.0 (21.7)[b,c] | <.001 |
| | | | | |
| Life satisfaction at wave -1, mean (SD), n=3535 | 10.3 (1.9)[a,c] | 16.1 (1.3)[a,b] | 18.8 (0.8)[b,c] | <.001 |
| | | | | |
| [a,b,c] Values with the same superscript are statistically significantly different at p <.05 | | | | |

**Supplementary Table S2.** Participant characteristics across categorized levels of leisure-time physical activity before retirement among 3535 participants providing information on life satisfaction, leisure-time physical activity and covariates before and after retirement.

| | **Level of leisure-time physical activity before retirement** | | | |
|---|---|---|---|---|
| **Total, n=3535** | **<14 METh/week** | **14-30 METh/week** | **30+ METh/week** | **p-value** |
| n (%) | 1323 (37.4%) | 1068 (30.2%) | 1144 (32.4%) | |
| Age, years, mean (SD) | 63.4 (1.4) | 63.4 (1.4) | 63.4 (1.4) | .587 |
| Women, n (%) | 1097 (82.9%) | 895 (83.8%) | 948 (82.9%) | .803 |
| Occupational background, n (%) | | | | . 251 |
| High | 421 (32.1%) | 387 (36.5%) | 386 (34.0%) | |
| Intermediate | 421 (32.1%) | 320 (30.2%) | 346 (30.4%) | |
| Low | 468 (35.7%) | 353 (33.3%) | 405 (35.6%) | |
| Married/cohabiting, n (%) | 940 (71.9%) | 746 (70.6%) | 800 (70.9%) | .758 |
| Suboptimal self-rated health, n (%) | 435 (32.9%)[a,c] | 240 (22.5%)[a,b] | 170 (14.9%)[b,c] | <.001 |
| Mobility limitations, n (%) | 305 (23.1%)[a,c] | 115 (10.8%)[a,b] | 51 (4.5%)[b,c] | <.001 |
| BMI, kg/m$^2$, mean (SD) | 28.4 (4.9)[a,c] | 26.4 (4.1)[a,b] | 25.5 (3.8)[b,c] | <.001 |
| METh/week at wave -1, mean (SD) | 7.69 (3.40)[a,c] | 20.79 (3.78)[a,b] | 45.30 (20.31)[b,c] | <.001 |
| Life satisfaction at wave -1, mean (SD) | 16.25 (2.83)[a,b] | 16.60 (2.45)[a] | 16.86 (2.30)[b] | <.001 |

[a,b,c] Values with the same superscript are statistically significantly different at p <.05

**Supplementary Table S3.** Participant characteristics by life satisfaction change group across retirement transition among 3371 participants providing full information on covariates.

| Life satisfaction across retirement transition, n=3371 | Stable Low n=133 | Low-increasing n=151 | | Intermediate-Decreasing n=83 | Stable Intermediate n=1271 | Intermediate-Increasing n=553 | | High-decreasing n=302 | Stable High n=878 |
|---|---|---|---|---|---|---|---|---|---|
| Women, % | 84.2% | 86.1% | | 85.5% | 80.3% | 85.0% | | 77.8% | 85.4% |
| Occupational background, % | | | | | | | | | |
| High | 30.1% | 27.2% | | 30.1% | 31.0% | 33.8% | | 34.1% | 42.1% |
| Intermediate | 37.6% | 36.4% | | 30.1% | 33.0% | 32.4% | | 28.2% | 26.7% |
| Low | 32.3% | 36.4% | | 39.8% | 36.0% | 33.8% | | 37.8% | 31.2% |
| Age, years, mean (SD) | | | | | | | | | |
| Wave -1 | 63.6 (1.4) | 63.3 (1.4) | | 63.7 (1.4) | 63.4 (1.5) | 63.2 (1.4) | | 63.6 (1.4) | 63.5 (1.4) |
| Wave +1 | 64.6 (1.4) | 64.3 (1.4) | | 64.7 (1.4) | 64.4 (1.5) | 64.2 (1.4) | | 64.6 (1.4) | 64.5 (1.4) |
| Married/cohabiting, % | | | | | | | | | |
| Wave -1 | 48.9% | 53.6% | | 67.5% | 69.6% | 73.6% | | 78.5% | 77.2% |
| Wave +1 | 45.9% | 54.3% | | 61.5% | 69.0% | 74.1% | | 79.5% | 78.0% |
| Suboptimal self-rated health, % | | | | | | | | | |
| Wave -1 | 54.1% | 49.0% | | 45.8% | 27.9% | 22.2% | | 14.2% | 11.3% |
| Wave +1 | 53.4% | 29.8% | | 45.8% | 23.8% | 12.8% | | 14.6% | 8.8% |
| Mobility limitations, % | | | | | | | | | |
| Wave -1 | 30.1% | 23.2% | | 19.3% | 14.6% | 11.4% | | 10.3% | 8.8% |
| Wave +1 | 28.6% | 21.2% | | 25.3% | 14.8% | 9.4% | | 10.9% | 8.2% |
| Body mass index, kg/m2, mean (SD) | | | | | | | | | |
| Wave -1 | 27.9 (6.1) | 27.7 (4.5) | | 27.7 (5.0) | 26.8 (4.3) | 26.6 (4.3) | | 26.7 (4.4) | 26.6 (4.7) |
| Wave +1 | 27.8 (5.3) | 27.8 (4.5) | | 27.8 (5.0) | 26.9 (4.4) | 26.6 (4.3) | | 26.7 (4.5) | 26.7 (4.7) |
| Physical activity, METh/week, mean (SD) | | | | | | | | | |
| Wave -1 | 18.7 (16.6) | 18.3 (14.9) | | 22.4 (14.6) | 23.0 (19.0) | 24.2 (19.3) | | 24.4 (21.4) | 26.6 (22.0) |
| Wave +1 | 19.5 (16.6) | 23.4 (19.8) | | 19.7 (14.5) | 24.6 (19.7) | 28.0 (19.8) | | 26.3 (21.3) | 29.2 (22.2) |

**Supplementary Table S4.** Percentages of categorized physical activity levels at wave -1 and wave +1 across life satisfaction change groups.

| **Life satisfaction across retirement transition n=3371** | **Stable Low n=133** | **Low-increasing n=151** | | **Intermediate-Decreasing n=83** | **Stable Intermediate n=1271** | **Intermediate-Increasing n=553** | | **High-decreasing n=302** | **Stable High n=878** |
|---|---|---|---|---|---|---|---|---|---|
| | | | | | | | | | |
| **Wave -1, %** | | | | | | | | | |
| <14METh/week | 50.4% | 48.3% | | 33.7% | 38.2% | 36.9% | | 38.1% | 32.2% |
| 14-30METh/week | 26.3% | 29.8% | | 30.1% | 32.1% | 30.0% | | 27.5% | 29.5% |
| 30+METh/week | 23.3% | 21.9% | | 36.1% | 29.7% | 33.1% | | 34.4% | 38.3% |
| | | | | | | | | | |
| **Wave +1, %** | | | | | | | | | |
| <14METh/week | 48.1% | 37.8% | | 45.8% | 35.2% | 27.7% | | 32.5% | 27.3% |
| 14-30METh/week | 30.1% | 33.8% | | 27.7% | 30.5% | 29.7% | | 28.5% | 28.3% |
| 30+METh/week | 21.8% | 28.5% | | 26.5% | 34.4% | 42.7% | | 39.1% | 44.4% |

**Supplementary Table S5.** Cumulative odds ratios (cOR) for higher activity category in wave +1 when compared to wave -1 across life satisfaction change groups with 95% confidence intervals (95% CI).

| **Life satisfaction across retirement transition n=3371** | **Stable Low n=133** | **Low-increasing n=151** | | **Intermediate-Decreasing n=83** | **Stable Intermediate n=1271** | **Intermediate-Increasing n=553** | | **High-decreasing n=302** | **Stable High n=878** | **Time*change, p-value** |
|---|---|---|---|---|---|---|---|---|---|---|
| **Unadjusted model** | **cOR (95% CI)** | | | | | | | | | .006 |
| cOR for higher activity at wave +1 vs. wave -1 | 1.04 (0.77 to 1.39) | 1.48 (1.10 to 1.98) | | 0.61 (0.39 to 0.96) | 1.18 (1.06 to 1.32) | 1.52 (1.29 to 1.78) | | 1.26 (1.01 to 1.58) | 1.28 (1.13 to 1.46) | |
| Comparison of cOR for higher activity at wave +1 vs. wave -1 | ref | 1.43 (0.94 to 2.16) | | 0.52 (0.33 to 0.83) | ref | 1.29 (1.06 to 1.56) | | 0.98 (0.76 to 1.27) | ref | |
| | | | | | | | | | | |
| **Model 1**[a] | **cOR (95% CI)** | | | | | | | | | .007 |
| cOR for higher activity at wave +1 vs. wave -1 | 1.00 (0.74 to 1.36) | 1.45 (1.07 to 1.96) | | 0.60 (0.38 to 0.95) | 1.15 (1.02 to 1.31) | 1.47 (1.23 to 1.76) | | 1.23 (0.97 to 1.55) | 1.23 (1.06 to 1.43) | |
| Comparison of cOR for higher activity at wave +1 vs. wave -1 | ref | 1.45 (0.96 to 2.19) | | 0.52 (0.33 to 0.83) | ref | 1.27 (1.05 to 1.55) | | 1.00 (0.77 to 1.30) | ref | |
| | | | | | | | | | | |
| **Model 2**[b] | **cOR (95% CI)** | | | | | | | | | .046 |
| cOR for higher activity at wave +1 vs. wave -1 | 1.01 (0.72 to 1.42) | 1.50 (1.08 to 2.08) | | 0.67 (0.40 to 1.12) | 1.19 (1.04 to 1.36) | 1.49 (1.23 to 1.81) | | 1.27 (0.99 to 1.63) | 1.28 (1.09 to 1.51) | |
| Comparison of cOR for higher activity at wave +1 vs. wave -1 | ref | 1.48 (0.94 to 2.35) | | 0.56 (0.33 to 0.95) | ref | 1.26 (1.02 to 1.55) | | 0.99 (0.75 to 1.31) | ref | |

[a] Adjusted for age, gender, occupational background, and marital status

[b] Adjusted age, gender, occupational background, and marital status, self-rated health, mobility limitations, and body mass index

**Supplementary Table S6.** Changes in leisure-time physical activity across retirement transition across life satisfaction change groups while using sample-specific cut-points for classifying life satisfaction.

| **Life satisfaction across retirement transition n=3371** | **Stable Low n=160** | **Low-increasing n=200** | | **Intermediate-Decreasing n=120** | **Stable Intermediate n=2377** | **Intermediate-Increasing n=257** | | **High-Decreasing n=125** | **Stable High n=132** | **Time*change, p-value** |
|---|---|---|---|---|---|---|---|---|---|---|
| **LTPA (METh/week)** | | | | | | | | | | |
| Unadjusted model | **Mean (95% CI)** | | | | | | | | | p=.028 |
| Change from wave -1 to wave +1 | 0.92<br>(-0.99 to 2.84) | 3.91<br>(1.59 to 6.24) | | -1.37<br>(-4.52 to 1.78) | 2.18<br>(1.39 to 2.96) | 4.21<br>(1.72 to 6.70) | | -0.10<br>(-3.47 to 3.27) | 4.40<br>(0.84 to 7.97) | |
| Mean difference in mean change | ref | 2.99<br>(-0.02 to 6.00) | | -3.55<br>(-6.80 to -0.30) | ref | 2.04<br>(-0.58 to 4.65) | | -4.50<br>(-9.41 to 0.41) | ref | |
| Model 1[a] | **Mean (95% CI)** | | | | | | | | | p=.027 |
| Change from wave -1 to wave +1 | 0.46<br>(-1.58 to 2.49) | 3.49<br>(1.07 to 5.91) | | -1.83<br>(-5.03 to 1.37) | 1.77<br>(0.70 to 2.84) | 3.80<br>(1.17 to 6.42) | | -0.45<br>(-3.88 to 2.99) | 3.96<br>(0.34 to 7.58) | |
| Mean difference in mean change | ref | 3.03<br>(0.03 to 6.04) | | -3.60<br>(-6.83 to -0.37) | ref | 2.02<br>(-0.59 to 4.64) | | -4.41<br>(-9.32 to 0.51) | ref | |
| Model 2[b] | **Mean (95% CI)** | | | | | | | | | p=.071 |
| Change from wave -1 to wave +1 | 0.56<br>(-1.57 to 2.69) | 3.12<br>(0.75 to 5.49) | | -1.34<br>(-4.54 to 1.85) | 1.90<br>(0.85 to 2.96) | 3.83<br>(1.21 to 6.45) | | 0.04<br>(-3.42 to 3.50) | 4.15<br>(0.54 to 7.77) | |
| Mean difference in mean change | ref | 2.56<br>(-0.48 to 5.60) | | -3.25<br>(-6.48 to -0.02) | ref | 1.93<br>(-0.69 to 4.54) | | -4.11<br>(-9.05 to 0.83) | ref | |

[a] Adjusted for age, gender, occupational background, and marital status

[b] Adjusted age, gender, occupational background, and marital status, self-rated health, mobility limitations, and body mass index

**Supplementary Table S7.** Changes in leisure-time physical activity across retirement transition across life satisfaction change groups while using five imputed data sets

| **Life satisfaction across retirement transition n=3535** | **Stable Low n=141** | **Low-increasing n=160** | | **Intermediate-Decreasing n=88** | **Stable Intermediate n=1341** | **Intermediate-Increasing n=579** | | **High-Decreasing n=314** | **Stable High n=912** | **Time*change, p-value[c]** |
|---|---|---|---|---|---|---|---|---|---|---|
| **LTPA (METh/week)** | | | | | | | | | | |
| Unadjusted model | **Mean (95% CI)** | | | | | | | | | .005 |
| Change from wave -1 to wave +1 | 0.47 (-1.27 to 2.20) | 4.12 (1.35 to 6.88) | | -2.91 (-6.34 to 0.51) | 1.55 (0.54 to 2.55) | 3.71 (2.17 to 5.25) | | 2.07 (-0.22 to 4.36) | 2.52 (1.18 to 3.86) | |
| Mean difference in mean change | ref | 3.65 (0.38 to 6.92) | | -4.46 (-8.03 to -0.89) | ref | 2.16 (0.32 to 4.01) | | -0.45 (-3.11 to 2.21) | ref | |
| Model 1[a] | **Mean (95% CI)** | | | | | | | | | .005 |
| Change from wave -1 to wave +1 | 0.11 (-1.76 to 1.98) | 3.86 (1.02 to 6.69) | | -3.21 (-6.70 to 0.29) | 1.26 (0.08 to 2.43) | 3.40 (1.67 to 5.13) | | 1.81 (-0.63 to 4.25) | 2.17 (0.61 to 3.73) | |
| Mean difference in mean change | ref | 3.75 (0.49 to 7.00) | | -4.46 (-8.03 to -0.90) | ref | 2.14 (0.29 to 3.99) | | -0.36 (-3.01 to 2.29) | ref | |
| Model 2[b] | **Mean (95% CI)** | | | | | | | | | .020 |
| Change from wave -1 to wave +1 | 0.07 (-1.94 to 2.09) | 3.55 (0.79 to 6.30) | | -2.59 (-6.17 to 0.99) | 1.35 (0.18 to 2.52) | 3.27 (1.53 to 5.01) | | 2.08 (-0.37 to 4.52) | 2.36 (0.81 to 3.91) | |
| Mean difference in mean change | ref | 3.47 (0.20 to 6.74) | | -3.94 (-7.60 to -0.28) | ref | 1.92 (0.06 to 3.79) | | -0.28 (-2.93 to 2.37) | ref | |

[a] Adjusted for age, gender, occupational background, and marital status

[b] Adjusted age, gender, occupational background, and marital status, self-rated health, mobility limitations, and body mass index

[c] Average p value from models with five imputed data sets

**Supplementary Table S8.** Changes in leisure-time physical activity across retirement transition across life satisfaction change groups among 2754 participants not reporting mobility limitations either before or after retirement.

| **Life satisfaction across retirement transition n=2754** | **Stable Low n=80** | **Low-increasing n=104** | | **Intermediate-Decreasing n=58** | **Stable Intermediate n=1019** | **Intermediate-Increasing n=465** | | **High-Decreasing n=258** | **Stable High n=770** | **Time*change, p-value** |
|---|---|---|---|---|---|---|---|---|---|---|
| **LTPA (METh/week)** | | | | | | | | | | |
| Unadjusted model | **Mean (95% CI)** | | | | | | | | | .057 |
| Change from wave -1 to wave +1 | 2.16 (-0.15 to 4.48) | 4.83 (1.89 to 7.77) | | -3.69 (-8.49 to 1.11) | 1.82 (0.63 to 3.02) | 3.96 (2.11 to 5.80) | | 2.36 (-0.23 to 4.96) | 3.03 (1.53 to 4.53) | |
| Mean difference in mean change | ref | 2.66 (-1.08 to 6.40) | | -5.52 (-10.46 to -0.57) | ref | 2.13 (-0.07 to 4.33) | | -0.67 (-3.66 to 2.33) | ref | |
| Model 1[a] | **Mean (95% CI)** | | | | | | | | | .060 |
| Change from wave -1 to wave +1 | 1.64 (-0.83 to 4.11) | 4.39 (1.34 to 7.43) | | -4.09 (-8.92 to 0.75) | 1.40 (0.05 to 2.74) | 3.54 (1.50 to 5.57) | | 1.98 (-0.81 to 4.76) | 2.58 (0.83 to 4.33) | |
| Mean difference in mean change | ref | 2.75 (-0.99 to 6.48) | | -5.48 (-10.42 to -0.54) | ref | 2.14 (-0.06 to 4.34) | | -0.61 (-3.59 to 2.38) | ref | |
| Model 2[b] | **Mean (95% CI)** | | | | | | | | | .086 |
| Change from wave -1 to wave +1 | 1.60 (-1.18 to 4.38) | 4.16 (1.16 to 7.17) | | -3.80 (-8.66 to 1.07) | 1.47 (0.13 to 2.81) | 3.53 (1.48 to 5.58) | | 2.18 (-0.61 to 4.97) | 2.79 (1.04 to 4.53) | |
| Mean difference in mean change | ref | 2.57 (-1.34 to 6.47) | | -5.26 (-10.23 to -0.30) | ref | 2.06 (-0.16 to 4.27) | | -0.60 (-3.59 to 2.39) | ref | |

[a] Adjusted for age, gender, occupational background, and marital status

[b] Adjusted age, gender, occupational background, and marital status, self-rated health, and body mass index
